# SARS-CoV-2 and Placenta: New Insights and Perspectives

**DOI:** 10.3390/v13050723

**Published:** 2021-04-21

**Authors:** Leonardo Resta, Antonella Vimercati, Gerardo Cazzato, Giulia Mazzia, Ettore Cicinelli, Anna Colagrande, Margherita Fanelli, Sara Vincenza Scarcella, Oronzo Ceci, Roberta Rossi

**Affiliations:** 1 Section of Pathology, Department of Emergency and Organ Transplantation (DETO), University of Bari “Aldo Moro”, 70124 Bari, Italy; leonardo.resta@uniba.it (L.R.); giulia.mazzia1@gmail.com (G.M.); anna.colagrande@gmail.com (A.C.); vincenza.scarcella@policlinico.ba.it (S.V.S.); oronzoruggiero.ceci@uniba.it (O.C.); roberta.rossi@policlinico.ba.it (R.R.); 2 Department of Biomedical Sciences and Human Oncology, Gynecologic and Obstetrics Clinic, University of Bari “Aldo Moro”, 70124 Bari, Italy; antonella.vimercati@uniba.it (A.V.); ettore.cicinelli@uniba.it (E.C.); 3 Medical Statistic, Department of Interdisciplinary Medicine, University of Bari “Aldo Moro”, 70124 Bari, Italy; margherita.fanelli@uniba.it

**Keywords:** SARS-CoV-2, pregnancy, COVID-19, placenta, transmission, outcomes, viruses

## Abstract

The study of SARS-CoV-2 positive pregnant women is of some importance for gynecologists, obstetricians, neonatologists and women themselves. In recent months, new works have tried to clarify what happens at the fetal–placental level in women positive for the virus, and different pathogenesis mechanisms have been proposed. Here, we present the results of a large series of placentas of Coronavirus disease (COVID) positive women, in a reference center for COVID-positive pregnancies, on which we conducted histological, immunohistochemical and electron microscopy investigations. A case–control study was conducted in order to highlight any histopathological alterations attributable to SARS-CoV-2. The prevalence of maternal vascular malperfusion was not significantly different between cases and controls (54.3% vs. 43.7% *p* = 0.19), whereas the differences with regard to fetal vascular malperfusion (21.1% vs. 4.2% *p* < 0.001) were significant. More frequent in cases with respect to controls were decidual arteriopathy (40.9% vs. 1.4% *p* < 0.0001), decidual inflammation (32.4% vs. 0.7% *p* < 0.0001), perivillous fibrin deposition (36.6% vs. 3.5% *p* < 0.0001) and fetal vessel thrombi (22.5% vs. 0.7% *p* < 0.0001). No significant differences in the percentage of terminal villous hyperplasia and chorioamnionitis were observed between the two groups. As the pandemic continues, these studies will become more urgent in order to clarify the possible mechanism of maternal–fetal transmission of the virus.

## 1. Introduction

At the end of December 2019, Chinese doctors in Wuhan, in the province of Hubei, China, started to report the first cases of an anomalous pulmonary infection not directly attributable to known infectious agents [1]. By the start of January 2020, the World Health Organization (WHO) had confirmed that the etiological agent in the cases of pneumonia was a new strain of Coronavirus, denominated SARS-CoV-2 (Severe Acute Respiratory Syndrome due to Coronavirus-2), and within just a few months, the pandemic still unfolding today had developed [1,2]. Despite the morphological and genomic resemblances to SARS-CoV-1, responsible for the Asiatic SARS epidemic in 2002–2004, and to MERS-CoV, associated with the Middle East Coronavirus respiratory syndrome, SARS-CoV-2 is much more contagious, although the mortality rate is actually lower [1,2,3,4]. In Italy, the first confirmed cases date back to the end of January, when two Chinese tourists from the province of Hubei, traveling to Rome, were found positive for SARS-CoV-2 [5]. Then, in February, after the first infections that developed in Codogno (Lombardy), many other infection foci emerged, firstly mainly in northern Italy, in the Lombard provinces of Brescia, Bergamo and Milan, but then spreading all over the peninsula [5,6]. By 11 February 2021, all the Italian regions had been infected by COVID-19 and more than two million positive cases had been recorded [7].

As regards placental disease in virus-positive pregnant women, only case reports or small, limited case series were reported in the first months of the pandemic [4,8,9,10]. However, as time passed, more and more cases of placental infection by SARS-CoV-2 were described, and there can be no doubt that with the progressive reduction in age of patients affected, the question of placental involvement and of potential maternal–fetal transmission has become an important matter of debate [10,11,12].

A study of the current literature seems to show that neonatal transmission is very rare, and that there are no specific SARS-CoV-2 histopathologic placental modifications observed in adverse perinatal outcomes, nor is there any evident greater risk of spontaneous abortion, preeclampsia, pre-term delivery or stillbirth [4,11,12]. However, few large case series have yet been reported owing to the obvious technical instrumental difficulties [13]. The present case–control study aimed to report the analysis of a large series of placentas from SARS-CoV-2-positive mothers observed at a COVID-19 reference center during the pandemic, and to compare them with a control group in order to highlight any histopathological alterations attributable to SARS-CoV-2. The research is documented by histopathological, ultrastructural and immunohistochemical findings, which are compared with other literature data.

## 2. Materials and Methods

### 2.1. Patients

The study was made of 83 placentas from 81 pregnant mothers (2 twin pregnancies) followed at the Gynecology and Obstetrics Operative Unit from 15 September 2020 to 31 January 2021, identified through electronic clinical records. All the women who presented during labor and delivery underwent testing with GeneXpert Dx Xpress SARS-CoV-2 RT-PCR (Cepheid) [14]. The analytical sensitivity and specificity of this test are reported by the manufacturers as 100% (87/87 samples) and 100% (30/30 samples), respectively, with a detection limit of 250 copies/mL or 0.0100 plaque-forming units per milliliter [15]. Positivity to the SARS-CoV-2 test was an independent criterion for the histopathologic analysis of the placentas. Among the positive SARS-CoV-2 group, twelve cases were excluded because they were related to unavoidable abortions due to maternal pathologies, which occurred in the 2nd trimester of the pregnancy, and to endo-uterine fetal deaths (EUFD) unrelated to SARS-CoV-2 positivity. The final sample included 71 placentas.

### 2.2. Controls

The SARS-CoV-2 group was compared with a control group of 142 placentas (1:2), selected from a population of pregnancy with physiological outcome, matched by gestational age and maternal age. Historical controls were selected from an archive of 500 placentas of women who had given birth between 2013 and 2018, of which 214 had a physiological fetal outcome. In accordance with the Amsterdam criteria, the parameters considered were: early maternal malperfusion, late maternal malperfusion, fetal malperfusion, placental infection/inflammation, villitis of unknown origin, delayed maturation of the villi; in addition, placental alterations (excluded from the Amsterdam criteria), including intravenous and chorangiotic hemorrhage, were taken into account. All records were retrieved from the electronic archives of our laboratory.

### 2.3. Procedure

The placentas were fixed in Formalin buffered at 10%, and photographs of the maternal and fetal surfaces were taken; they were then weighed, sampled and examined along the cut surface. The samples obtained included 2 rolls of amnio-chorial membrane, at least 2 samples from the umbilical cord, 3 from the maternal surface, 2 full-thickness sections and representative samples of any lesions presents. All samples were subjected to routine treatment, inclusion, 5 µm sectioning and hematoxylin–eosin staining (H&E). They were observed with an Olympus BX-51 Optical Microscope equipped with the Olympus DP80 image acquisition system. To randomly chosen sections of 51 placentas, the anti-SARS-CoV-2 spike S1 glycoprotein monoclonal antibody, Thermofisher, Rabbit, was added, at pH 6, diluted 1:800, and the antigenic unmasking heat-induced citrate buffer epitope retrieval, for enzymatic immunohistochemical (IHC) analysis. In addition, electronic microscopy analysis was performed for 30 of the 83 placentas. At the moment of delivery, random placental parenchyma samples were immediately fixed in 2.5% Gluteraldehyde for 4 h at 4 °C, and after overnight immersion in phosphate buffer, post-fixed with Osmium Tetroxide in PBS for 2 h at a temperature of 4 °C. The prepared samples were processed for inclusion in araldite epoxy resin (M) CY212 (TAAB, Aldermason, UK). Semifine sections 0.5 µm thick were stained with Toluidine Blue for microscopic analysis. Ultrafine sections were mounted on nickel grilles with uranium acetate and lead citrate contrast. The semifine sections were observed with a Nikon photomicroscope equipped with a Nikon Coolpix DS-U1 Digital Camera (Nikon Instruments SpA, Calenzano, Italy). The ultrafine sections were observed with a Morgagni 268 electron transmission microscope (FEI Company, Naples, Italy). All cases were examined independently under double-blind conditions by two pathologists with expertise in the field of perinatal pathology, to confirm the diagnoses.

### 2.4. Statistical Analysis

A preliminary description of maternal features in the SARS-CoV-2 group and control group was made, comparisons between means were performed with Student’s t test for independent groups, and comparisons between percentages were made via chi-square test. The placental findings were compared by chi-square test. To correct for the potential presence of type I errors induced by multiple testing, all results were false discovery rate (FDR) corrected with alpha = 0.05. Quantities are reported as mean ± standard deviation. Categorical data are reported as frequencies and percentages. Statistical analysis was performed by means of SAS Software 9.4 (https://support.sas.com/software/94/index.html).

## 3. Results

All the SARS-CoV-2-positive pregnant women who presented to the Gynecologic and Obstetrics Clinic in the period between 15 September 2020 and 16 January 2021 were enrolled in the study. Of the 83 placentas in the SARS-CoV-2 -positive group, 6 were from unavoidable abortions due to maternal disease that developed in the second trimester of pregnancy and 6 from endo-uterine fetal deaths (EUFD) in the third trimester; all these placentas were excluded from the statistical analyses. The final sample included 71 placentas; 62 women had delivered at term (37–42 weeks) and 7 preterm (≤37 weeks). The mean gestational period was 38.5 ± 2.9 (20–42) weeks. The gravidae were aged 19 to 46 years, with a mean age 33.1 ± 6.1 years; 37 had a spontaneous vaginal delivery, 32 underwent cesarian section, urgent or elective; 31 were primiparous, 25 at their second pregnancy and 13 multiparous. No prior disease before or during pregnancy was reported by 54 women, while 3 were carriers of the methylenetetrahydrofolate-reductase gene (MTHFR), 1 of which was in association with a PAI-1 deficit, 4 were in treatment for hypothyroidism, 5 were affected by gestational diabetes treated with a special diet (in 1 case the metabolic disease was associated with hypertension and in 1 with allergic asthma), and 6 women were affected by hypertension; finally, 2 women had developed hepatogestosis. All these women were positive for SARS-CoV-2 at the moment of delivery; 42 patients (60.9%) were asymptomatic while 24 (34.8%) had a flu-like syndrome with one or more of the following symptoms: slight fever, headache, cough, myalgia, anosmia and ageusia. Of these, 13% required treatment. Finally, three (4.3%) patients had moderate/severe symptoms (two patients needed oxygen in cannula ventilation and one patient mechanical non-invasive ventilation). The Apgar scores of liveborn infants at 1 min were 8 or 9. All Apgar scores at 5 min were 9 or 10. No neonatal deaths occurred. All the infants were negative for SARS-CoV-2 at nasopharyngeal and/or pharyngeal swab.

The 71 placentas from SARS-CoV-2 -positive mothers were compared with 142 control placentas matched by gestational age and maternal age. The maternal and pregnancy features are reported in Table 1.

In accordance with the matching, there were no significant differences in maternal and gestational age between SARS-CoV-2-positive and control mothers (*p* > 0.05). Maternal diseases were equally distributed in the two groups. PROMs were significantly less prevalent in the cases than the controls (8.4% vs. 23.9% *p* = 0.017), as were IUGR (0% vs. 12.7% *p* = 0.003) and oligohydramnios (1.4 vs. 9.2 *p* = 0.05). There were no differences for polyhydramnios (*p* = 0.61). Apgar scores at 1 min and 5 min were not significantly different (*p* = 0.83).

**Figure 1:**
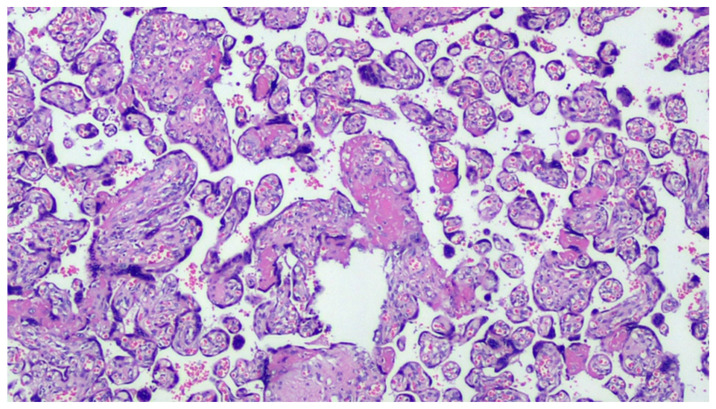
COVID-19-positive mother placenta. Terminal chorionic villi with poor vascular component (distal hypoplasia of the villi due to early maternal malperfusion) with increased syncytial nodes. Some villi show a deposition of fibrin in the intervillar space with progressive reduction of the villi (H&E, Hematoxylin and Eosin, 100×).

**Figure 2:**
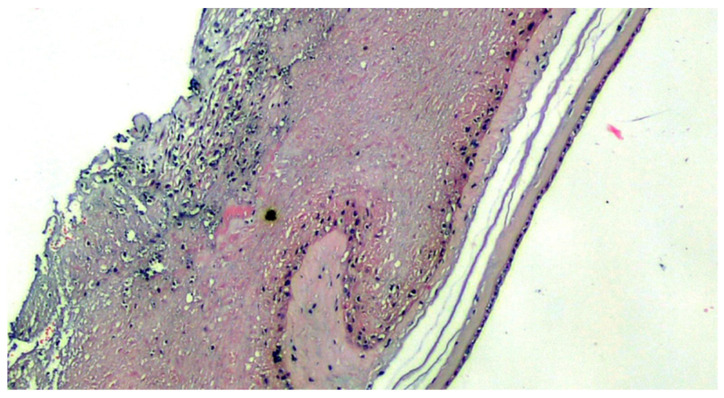
Amnio-chorionic membranes characterized by the presence of maternal neutrophilic granulocytes that infiltrate the sub-amniotic chorion starting from the maternal blood (H&E, 100×).

**Figure 3:**
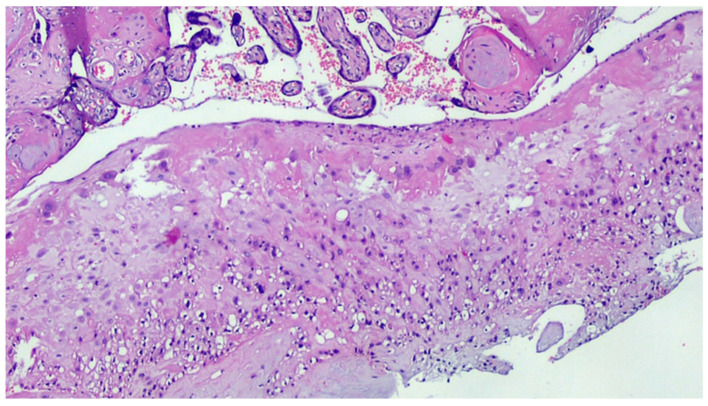
Basal deciduitis with a little trophoblastic component (superficial implant) and with few inflammatory cells (H&E, 200×).

**Figure 4:**
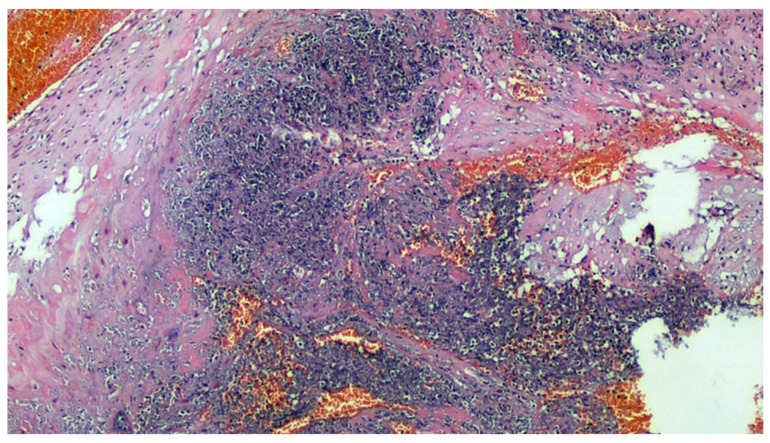
Deciduitis with large foci of necrosis and massive infiltration of mainly granulocytic inflammatory elements (acute deciduous, H&E, 200×) in the placenta of a COVID-positive mother.

**Figure 5:**
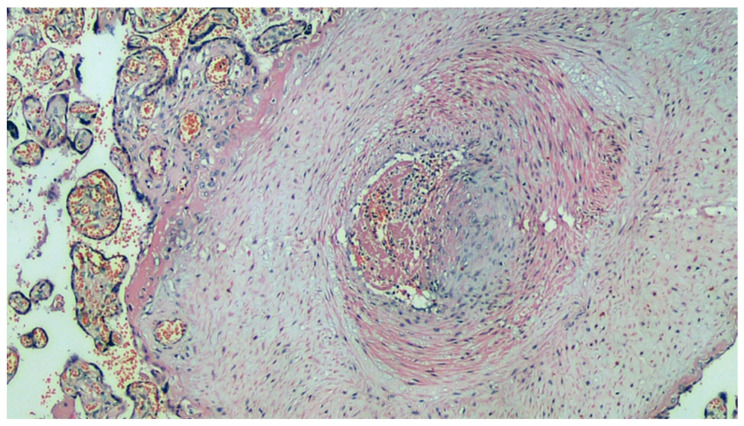
Histological section of the main villus: the artery has a thickened muscular wall, marked intimal fibrous thickening (probable organization of arterial thrombus), clear reduction of the lumen, and recent thrombosis of the residual lumen. H&E, 200×.

**Figure 6:**
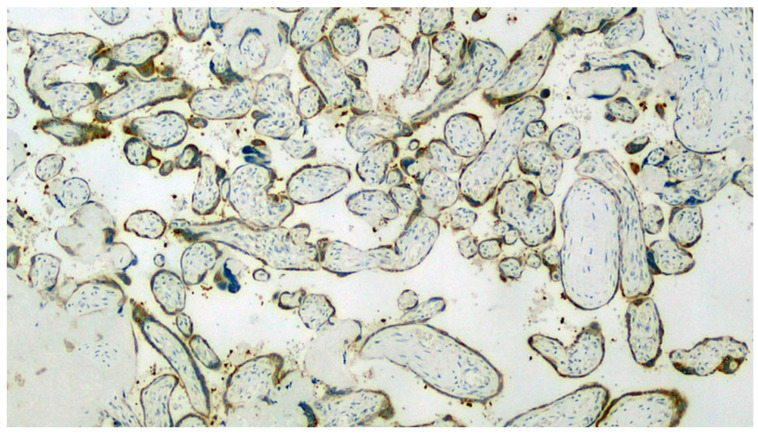
Section set up for immunohistochemistry investigation using the Sars-CoV-2 anti-spikes glycoprotein antibody. Note the widespread involvement of brown-colored syncytiotrophoblast (Immunohistochemistry, IHC, 100×).

**Figure 7:**
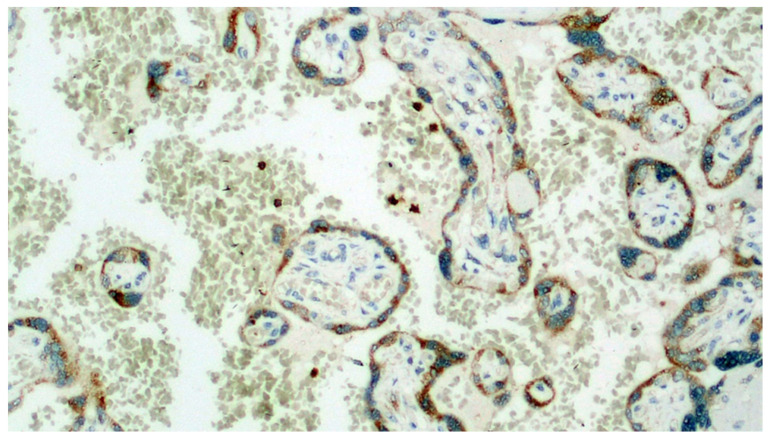
Detail of the previous image. In addition to the positivity expressed by the trophoblast, a very intense positivity is observed in the leukocytes of the maternal blood (IHC, 400×).

**Figure 8:**
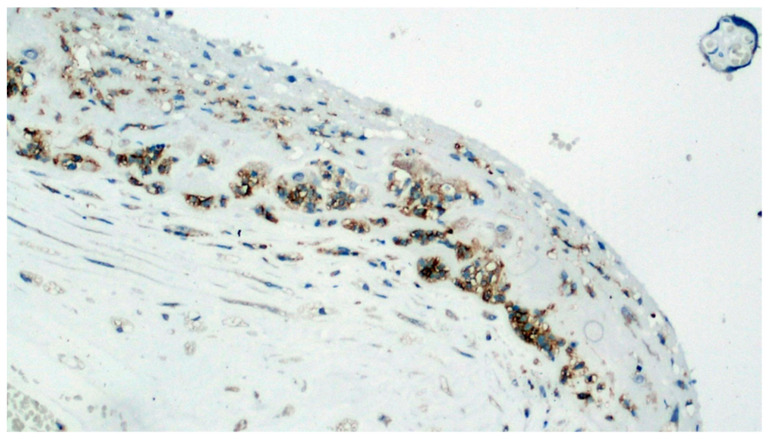
Expression of viral antigen in stromal cells of the basal decidua (IHC, 400×).

**Figure 9:**
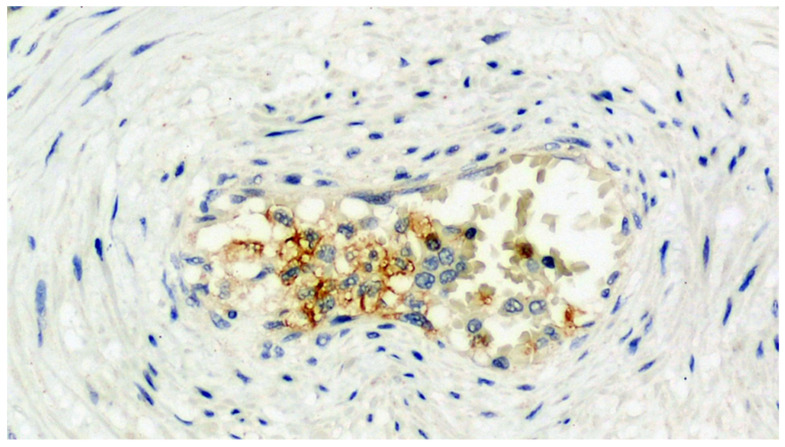
Detail of a small vessel of an 8th order main villus characterized by the expression of a spike glycoprotein antigen on endothelial cells degenerated during thrombosis (IHC, 400×).

The mean placental weight was not significantly different between the two groups (*p* = 0.48). No differences in the percentage of maternal vascular malperfusion were observed in the cases compared to controls (54.3% vs. 43.7% *p* = 0.19), whereas the differences with regard to fetal vascular malperfusion (21.1% vs. 4.2% *p* < 0.001) were significant. The same applied for decidual arteriopathy (40.9% vs. 1.4% *p* < 0.0001), decidual inflammation (32.4% vs. 0.7% *p* < 0.0001), perivillous fibrin deposition (36.6% vs. 3.5% *p* < 0.0001) and fetal vessel thrombi (22.5% vs. 0.7% *p* < 0.0001). In contrast, a lower percentage of villous hypervascularization (12.7% vs. 34.5% *p* < 0.001) was observed in the SARS-CoV-2-positive group compared to controls (Figure 1, Figure 2, Figure 3, Figure 4 and Figure 5). No significant differences in the percentage of terminal villous hyperplasia and chorioamnionitis were observed between the two groups (Table 2). The anti-SARS-CoV-2 spike-S1 glycoprotein antibody’s results were significantly different, with 33/51 cases (65%) of diffuse positivity throughout the examined section and 18/51 cases (35%) of localized positivity, the expression being prevalent in the cytoplasm of the villi trophoblasts. We also observed positivity in 13/51 (25%) cases in the endothelium of the villi capillaries in sites of thrombosis; 14/51 cases (28%) showed positivity in the maternal decidual cells and in the intervillous histiocytes from maternal blood (Figure 6, Figure 7, Figure 8 and Figure 9).

**Figure 10:**
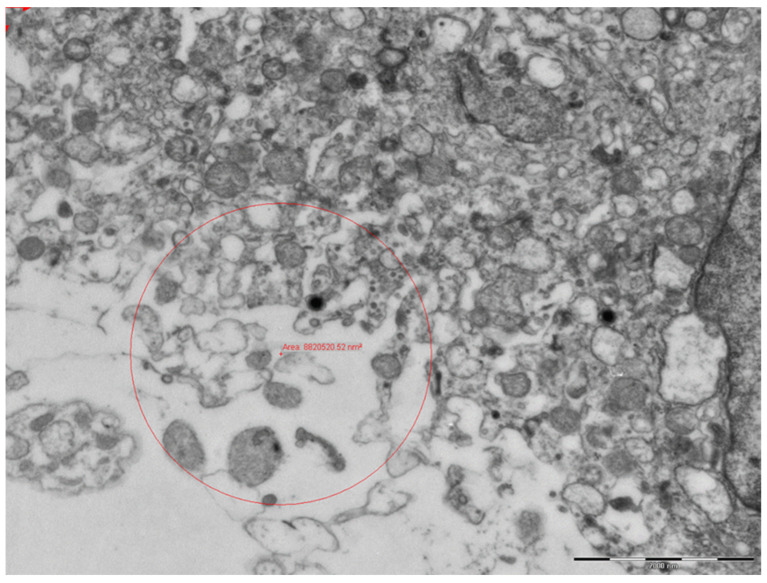
Photomicrograph of syncytium trophoblast cytoplasm. In addition to the microvillary projections, the cell has numerous secretory vacuoles, mitochondria and electrondense lysosomes (11,000×).

**Figure 11:**
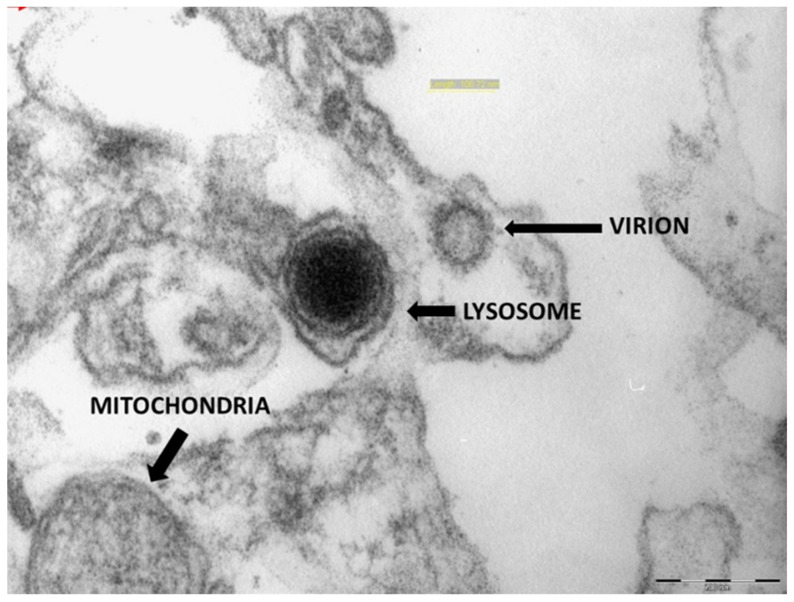
Strong magnification of a spherical particle of 106,720 nm with spicular electron-dense projections next to a strongly electrondense lysosomal formation (71,000×).

Electron microscopy showed signs of endothelial damage in the fetal vessels, with endothelial hypertrophy and a reduced lumen. In the cytoplasm of the trophoblasts of some cells, circular formations with a 100–130 nm diameter were observed, with peripheral electron dense spicules, which are likely viral particles (Figure 10 and Figure 11).

## 4. Discussion

In recent months, various reports have been published describing studies of the placentas of mothers affected by SARS-CoV-2. Some case reports and case series have aimed to throw light on the pathophysiologic aspects of SARS-CoV-2 infection in pregnant women [11,12,13,16]. These studies were focused in particular on the maternal outcomes of patients with symptomatic disease; maternal death, stillbirth and neonatal death were reported to occur in about 1% of the cases [17]. The risk of SARS-CoV-2 positivity in a newborn from a mother admitted to hospital with symptomatic disease is about 2.5% [17]. Few works in the literature have reported solid evidence of vertical transplacental transmission [12,13,16,18].

Allotey J et al. suggest that pregnant women with symptomatic SARS-CoV-2 infection are less likely to present with fever and myalgia, but are more likely to need intensive care, ventilation, and have a higher risk of pre-term delivery [19,20]; moreover, death occurs in a small number of cases that are COVID-related [21,22,23,24]. Among the 71 placentas we analyzed, there were no cases of the maternal–fetal transmission of SARS-CoV-2, and all the newborns were in good health at birth, with similar APGAR scores at 1 min and 5 min to those of the controls (*p* = 0.83). The cases of unavoidable abortion in the second trimester (*n* = 6) were linked to diseases not correlated with a viral infection in course, such as chromosomal alterations and maternal conditions such as systemic lupus erythematosus (SLE) and diabetes, which are in themselves often associated with a greater spontaneous abortion rate [25]. In the same way, the six endouterine fetal deaths were attributable to maternal conditions (pre-eclampsia and diabetes) that, in our opinion, could not be correlated with the infection [25,26]. As can be seen in Table 1, the placental alterations we observed are only partially comparable to those in the control population, with signs of maternal vascular malperfusion (MVM) being observed in both cohorts. In this sense, despite what was declared by Shanes [27] and Menter [13], we believe that the vascular modifications of villi considered to be signs of MVM can also be seen as functional adaptation phenomena in many cases, leading to a favorable neonatal outcome [28]. It is easier to correlate some of the alterations in the patients group to SARS-CoV-2 infection, such as the presence of thrombi in the utero–placental arteries, of deciduitis foci with tissue necrosis, and of an inflammatory infiltrate, which are likely correlated to virus-mediated damage, as pointed out by Baergen [29]; these were more frequent in the virus-infected patients than the controls. These latter findings were also confirmed by Prabhu et al. [30]. Phenomena such as fetal vascular malperfusion (FVM) and thrombosis, particularly of small caliber vessels of the main sixth or eighth order villi, can be correlated to SARS-CoV-2. Intervillous fibrin deposits are reported to be a very common observation in SARS-CoV-2-positive women, as described by Chen et al. [31]. This finding seems to be related more to an immuno-mediated outcome of maternal origin, rather than being a sign of true maternal malperfusion. [27,29]. It is possible that immunological stimulation of the mother, as well as trophoblasts damage, may be the underlying cause of the greater intervillous fibrin deposits in SARS-CoV-2-positive placentas. Some authors [12,21,22,24] have described the presence of intervillous histiocytes, interpreted as a sign of perivillitis. In our experience, perivillitis with a significantly elevated number of histiocytes was observed in only three cases, whereas the immunohistochemistry studies showed strong positivity for the anti-SARS-CoV-2 spike glycoprotein antibody in maternal white blood cells. It is important to note that several patients showed alterations normally attributable to maternal diseases that have nothing to do with viral infection, such as diabetes or latent hypertension, aspecific inflammatory reactions of the membranes, and non-specific unreactive villitis. However, the immunohistochemical studies showed the presence of the viral antigen in many maternal cells, particularly deciduous stromal and endothelial cells, and in perivillous inflammatory cells. This issue has already been pointed out by Patanè et al. [23]; indeed, the positivity observed in syncytiotrophoblasts could be interpreted as a barrier effect of these cells between the maternal and the fetal compartments. Only in rare cases was endothelial positivity in the fetal capillaries associated with thrombosis. The vascular damage that can occur is demonstrated by the frequent aspects of endothelial hyperplasia of the fetal capillaries and the reduced lumen that we observed. The finding, through electron microscopy, of structures compatible with the virus in syncytial cells, associated with gross vacuolization of the cytoplasm, confirms the positivity of this cell layer in terms of immunohistochemistry, as well as justifying the trophoblast necrosis phenomena described by some authors, and has been indicated as the cause of maternal–fetal transmission of the virus [32,33].

## 5. Conclusions

Our study, conducted on a large number of placentas, shows that in cases of SARS-CoV-2-positive pregnant women without transmission of the disease to the fetus, the placentas are largely unaffected by the inflammatory process. However, there are some more frequent characteristics in the placentas of infected women, in particular, maternal thrombosis and deciduous, increased intervillous fibrin, and, in rare cases, fetal thrombosis. The immunohistochemical investigation demonstrates positivity for the anti-SARS-CoV-2 spike glycoprotein antibody both among maternal cells (including inflammatory intervillary cells) and in the trophoblast, and rarely in the endothelium. The ultrastructural investigation demonstrated both the suffering of fetal endothelia and the presence of particles attributable to SARS-CoV-2 in the trophoblast, in conjunction with its degeneration.

As the pandemic continues, these studies will become more urgent for clarifying the possible mechanism of the maternal–fetal transmission of the virus.

## Figures and Tables

**Table 1:** Maternal and pregnancy features in SARS-COV-2 and control groups.

Maternal and Pregnancy Features	SARS-COV-2 Group	Control Group	Uncorrected *p* Values	FDR Corrected *p* Values
*Maternal age* (years), means ± sd (range)	33.1 ± 6.1 (19–46)	33.1 ± 5.7 (16–46)	0.97	0.97
*Gestational age* (week), means ± sd (range)	38.5 ± 2.9 (20–42)	38.9 ± 1.8 (32–42)	0.24	0.83
*Apgar score 1 min*, mean (sd)	9 (0.7)	9 (0.9)	0.62	0.83
*Apgar score 5 min*, mean (sd)	10 (0.5)	10 (0.4)	0.54	0.83
*Primiparous*, *n* (%)	31 (44.9)	97 (68.3)	0.0021	0.008
*Cesarean Section*, *n* (%)	32 (46.4)	124 (87.3)	<0.0001	0.0001
*Diabetes*, *n* (%)	5 (7)	11 (7.7)	0.55	0.61
*Hypertension*, *n* (%)	6 (8.6)	15 (10.6)	0.63	0.63
*Thyroid dysfunction*, *n* (%)	4 (5.8)	7 (4.9)	0.53	0.61
*Other pathology*, *n* (%)	3 (4.3)	2 (1.4)	0.20	0.37
*PROM*, *n* (%)	6 (8.6)	34 (23.9)	0.0063	0.017
*IUGR, n* (%)	0 (0)	18 (12.7)	0.0005	0.003
*Polyhydramnios, n* (%)	1 (1.4)	4 (2.8)	0.46	0.61
*Oligohydramnios, n* (%)	1 (1.4)	13 (9.2)	0.02	0.05

FDR correction was performed separately for means comparisons and for proportion comparisons. *n* = number of cases; sd = standard deviation.

**Table 2:** Placental findings in SARS-COV-2 and control groups.

Placental Finding	SARS-COV-2 Group (71 Cases)	Control Group (142 Cases)	Uncorrected *p* Values	FDR-Corrected *p* Values
*Weight* (grams), means ± sd (range)	515 ± 84 (240–760)	499.2 ± 176.6 (130–1020)	0.48	0.48
*Maternal malperfusion*, *n* (%)	38 (54.3)	62 (43.7)	0.15	0.19
*Decidual arteriopathy*, *n* (%)	29 (40.9)	2 (1.4)	<0.0001	<0.0001
*Fetal malperfusion*, *n* (%)	15 (21.1)	6 (4.2)	<0.0001	<0.0001
*Decidual inflammation*, *n* (%)	23 (32.4)	1 (0.7)	<0.0001	<0.0001
*Perivillous fibrin deposition*, *n* (%)	26 (36.6)	5 (3.5)	<0.0001	<0.0001
*Terminal villous hyperplasia n* (%)	14 (19.7)	30 (21.1)	0.81	0.81
*Villous hypervascularization*, *n* (%)	9 (12.7)	49 (34.5)	0.0007	0.0011
*Thrombi in fetal vessels*, *n* (%)	16 (22.2)	1 (0.7)	<0.0001	<0.0001
*Chorioamnionitis*, *n* (%)	5 (7)	7 (4.9)	0.37	0.41

FDR correction was performed separately for means comparisons and for proportion comparisons. *n* = number of cases.
